# Health care utilization among individuals who die by suicide as compared to the general population: a population-based register study in Sweden

**DOI:** 10.1186/s12889-022-14006-x

**Published:** 2022-08-25

**Authors:** Elisabeth Bondesson, Tori Alpar, Ingemar F. Petersson, Maria E. C. Schelin, Anna Jöud

**Affiliations:** 1 Faculty of Medicine, Department of Clinical Sciences Lund, Division of Orthopaedics, Lund University, Lund, Sweden; 2 Department of Neurosurgery and Pain Rehabilitation, Skåne University Hospital, Lund, Sweden; 3 Faculty of Medicine, Department of Laboratory Medicine, Division of Occupational and Environmental Medicine, Lund University, Lund, Sweden; 4 Department of Research and Education, Skåne University Hospital, Lund, Sweden; 5 Institute for Palliative Care, Lund University and Region Skåne, Lund, Sweden

**Keywords:** Suicide, Health care utilization, Electronic health record, Mental disorders, Population-based, Register, Epidemiology, Skåne healthcare register, Cause of death register

## Abstract

**Background:**

Globally, 700 000 people die every year by suicide. Health care consultation patterns the period before suicide could be one potential way to identify people at risk for suicide. Therefore, this study examines health care patterns up to one year prior to the suicide by age, sex and prior diagnoses and specifically investigates if and how this differs from the general population of Skåne, Sweden.

**Methods:**

This cohort study includes all individuals, aged 15 and older, that died by suicide in Region Skåne, Sweden from 2004 to 2015 (n = 1653). The individuals were identified through the Cause of death register and then linked to the Skåne healthcare register. Health care data was analyzed as proportions consulting different types of health care the month and year preceding the suicide, we also investigated the impact of age, sex and the occurrence of prior psychiatric and pain diagnoses. Additionally, we compared the proportion of consulting care among the suicide victims and the general population of Skåne.

**Results:**

In the month before their death, 53% of the suicide victims had any health care consultation, compared with 20% in the general population of Skåne, a given month (*p* < 0.0001). The corresponding figures for the year prior to suicide was 86% among those who died by suicide, compared to 69% in the general population of Skåne, a given year (*p* < 0.0001). Women, and those having a documented history of psychiatric diagnosis were more likely to have health care consultations in the month and year preceding suicide (*p* < 0.001), compared to men and suicide victims without a history of psychiatric disease. Older adults that died by suicide, were less likely to consult psychiatric care compared to the younger suicide victims (*p* < 0.001).

**Conclusion:**

A majority of the suicide victims consulted health care in the near time before death and the proportion of seeking health care was significantly higher than in the general population of Skåne and higher among female suicide victims as compared to males. Alternative preventive screening measures should be considered for individuals consulting health care, especially for older people and individuals outside the psychiatric care.

## Background

Suicide is a significant public health problem throughout all stages of life. With 700,000 deaths each year, suicide is among the primary causes of premature death worldwide [1], particularly among men and younger individuals. In Sweden, approximately 12–17 suicides per 100 000 individuals occur yearly, which is a bit higher as compared to the world age-standardized rate of 9 per 100 000 [1, 2].

Risk factors and causal mechanisms underlying suicide are complex and not completely understood. However, an underlying documented psychiatric disorder is an important risk factor for suicidal behavior [3]. The most common underlying mental illness is mood disorders, such as depression and bipolar disorder but also anxiety disorders, substance use disorders and schizophrenia are common [4–9]. Even though the link between mental disorders and suicide risk is very strong, for many patients it is often a life crisis such as marital breakups or economic problems that trigger the acute suicide risk [10]. Moreover, the single most important risk factor for suicide in the general population is a prior suicide attempt [1, 5].

Other risk factors include male sex, low socioeconomic status [3] and family history of suicide [11]. Various somatic diseases also act as risk factor, especially if the condition affects the physical function such as amyotrophic lateral sclerosis (ALS), Multiple sclerosis (MS) and different types of cancer [6, 12, 13]. Further, individuals with non-cancer pain conditions also have elevated suicide risks [14] and chronic pain itself, regardless of type, is an important independent risk factor for suicidality [15]. Additionally, it has been shown that pain and mental illness have a bidirectional influence, of similar magnitude, on one another [16].

There are interventions, psychological and/or pharmacological, that have been shown to be effective in reducing rate of suicide ideation, attempt and completed suicide [17–19], but the crucial thing is to identify individuals with suicidal ideation, those in need for these interventions. One potential arena for this identification is within the health care.

Studies from countries around the world, mainly conducted in Western European and North American countries, have shown that individuals who die by suicide do consult health care to a large extent the period before the death [20–24]. This provides a unique opportunity for intervention. However, to strengthen health care staff’s possibility to recognize and identify patients at risk for suicide, their pattern of consultation needs to differ from that of the rest of the population. A few studies have shown that health care use is more common across different health care settings for individuals who later die by suicide compared to matched individuals in the general population [4, 8, 22, 25]. For example, two US studies of both youths (including 910 suicides) and adults (including 2674 suicide) have shown that a greater proportion of suicide victims than controls use health care services. Odds ratios for any type of health care visit one month before the index date were 2.87 among youths and adjusted odds ratios were 2.67 among adults [4, 8]. From a study conducted in Wales that included 1721 suicide cases, an odds ratio of 2.7 was reported when comparing suicide victims and controls and their visits to any health care setting the last month before suicide [25]. Further, a Norwegian study including 4926 suicide victims reported that contact with GPs in primary care prior to suicide is common in both sexes. Risk ratios for any consultation last month were 2.23 among men and 2.05 among women when comparing suicide victims and population controls [22]. However, in some studies individuals with lower socioeconomic status, e.g. uninsured are not included [4, 8, 26] and other studies only cover specific health care settings, e.g. primary care [22, 27]. Thus, the extent to which the pattern of health care contacts among suicide victims’ deviates from those of the general population remains to be determined. Because health care systems are organized differently between countries results from one country cannot automatically be generalized to other countries and therefore country specific data is needed as a complement.

Therefore, the aim of this register-based study is to investigate health care consultation patterns across sex, age and prior diagnoses among suicide victims in the period from 2004 to 2015. Additionally, this pattern is compared to the pattern of consultation in the underlying general population in Skåne (*n* = 1.4 million inhabitants).

## Methods

This study used the total population of Region Skåne. This is in the southernmost part of Sweden and has a population of 1 377 827 million (2019). The region holds 13% of the national population and corresponds demographically to the whole of Sweden [28]. Sweden has universal health care i.e. a publicly funded health care system with all residents having access to health care for an annual small fee ($121, June 2022). No referral is necessary for access to primary care or outpatient specialized care. The health care system is decentralized and administered by 21 geographical regions with full responsibility for organizing, delivering and documenting all health care delivered in the region.

### Data sources

Two population-based registries were used—the Swedish cause of death register and the Skåne healthcare register.

The Swedish cause of death register, held by the National Board of Health and Welfare, is a high quality, virtually complete register of all deaths in Sweden since 1952 [29]. It is mandatory to register all deaths and the registration is based on the International Classification of Diseases, 10th revision. The register has been validated and is considered to have high coverage and high-quality data [29].

The Skåne healthcare register is an administrative health care database that has been running since the late 1990s within Region Skåne [30]. Each health care consultation in the region generates data entries by the health care provider that are then automatically transferred to the Skåne healthcare register, which thus contains data from all consultations to primary care, outpatient specialized care and inpatient care. The register has been validated and considered to be a reliable data source [31].

### Study population

All individuals from 15 years of age, residing in Skåne who died by suicide from 1 January 2004 to 31 December 2015 were identified from the Swedish cause of death register. In total, 1,653 individuals were identified.

For the suicide victims, death records were linked to health care records using the personal identity number given to each Swedish resident [32]. The general population i.e. those aged 15 years or older living in the region annually between 2004–2015 were retrieved from Statistics Sweden [33] and aggerated data on the number of unique individuals seeking health care was retrieved from the Skåne healthcare register.

### Definitions

Diagnoses are classified according to the Swedish translation of International Classification of Diseases and Related Health Problems (ICD) 10 system. Suicide deaths were defined as death caused by ICD-10 codes X60-X84 (Intentional self-harm), in line with the official cause of death statistics.

Prior psychiatric diagnosis was defined as having at least one ICD-10 diagnosis within the F chapter (Mental and behavioral disorders) recorded, by any health care professional at any type of consultation the month (30 days) and year (365 days) prior to suicide.

Prior pain diagnosis was defined as having at least one ICD-10 diagnosis of abdominal pain, headache, back/neck pain, joint pain/myalgia, pain not specified in primary care, or persistent pain recorded (ICD-10 codes ‘R10’, ‘G43’, ‘G44’, ‘R51’, ‘M54’, ‘M25.5’, ‘M79.1’, ‘M79.6’ ‘R52- ‘, ‘R52.9’, ‘M79.7’, ‘R52.1’, ‘R52.2’ or ‘F45.4’ including all subcategories) the month and year prior to suicide.

Type of health care was defined based on classifications of health care into three categories: *Any health care* defined as all types of health care, *Psychiatric care,* specifically defined as consultation within psychiatric care (this includes both inpatient and outpatient psychiatric care) and *Primary care* (only consultations in Primary care).

### Study variables

We calculated consultation rates as proportions (percentage) of individuals that consulted any health care and by type of health care (primary and psychiatric). The analysis was done for one month and one year prior to the suicide. Additionally, we also calculated the rate of individuals with prior psychiatric and pain diagnosis, the month and year prior to suicide.

For the general population of Skåne, corresponding rates were calculated. Annual population figures were retrieved from Statistics Sweden and served as denominators. Aggregated monthly and yearly number of consultations for years 2004 to 2015 (total and by different type of health care) were retrieved from the Skåne healthcare register and served as numerators. Four age groups were generated according to the age of the suicide victims in the year of death (i.e. 15–24, 25–39, 40–54, 55–69, and 70 years and older) and the same age groups were created for the general population of Skåne during 2004–2015.

### Statistical analyses

The proportion (percentage) of individuals consulting health care was computed and compared between suicide cases and the general population of Skåne in the same ages as the group of suicide victims. The analyses were done in total and stratified by sex and age, for the three types of health care categories, using MedCalc (MedCalc Software Ltd. Comparison of proportions calculator. https://www.medcalc.org/calc/comparison_of_proportions.php (Version 20.019; accessed September 29, 2021).

Within the group of suicide cases, chi-square tests were used to compare health care utilization, for the three types of health care categories, in total and by categories (age, sex, prior psychiatric and pain diagnosis) within one month and one year prior to death.

Cochrane-Armitage test for trend was done to investigate if the change over time was statistically significant. Analyses within the group of suicide cases were conducted using SAS, version 9.4 (SAS Institute Inc), with significance set at 2-sided *P* < 0.05.

## Results

In total, 1653 individuals, aged 15 and older that died by suicide between 2004–2015 were identified. The majority were men (69%) and it was common to have a documented psychiatric diagnosis (55%) the year prior to suicide. The largest number of suicides occurred in individuals aged 55–69 (26%) followed by those aged 40–54 (25%) (Table 1).

**Table 1:** Demographics for individuals dying by suicide (*n* = 1653), and the general population of Skåne (*n* = 1 022 423), 2004–2015

	Individuals dying by suicide	Population ^a^
Age		
Mean (SD)	54 (19.6)	N/A
Age categories % (n)		
15–24	9 (144)	15 (157 466)
25–39	17 (277)	24 (245 726)
40–54	25 (420)	24 (241 650)
55–69	26 (432)	21 (218 266)
≤ 70	23 (380)	16 (159 315)
Sex % (n)		
Male	69 (1143)	49 (502 114)
Female	31 (510)	51 (520 309)
Psychiatric diagnosis % (n) ^b^		
Yes	55 (902)	N/A
No	45 (751)	N/A
Pain diagnosis % (n) ^c^		
Yes	21 (350)	N/A
No	79 (1303)	N/A

^a^ Mean values for the years 2004–2015

^b^ At least one recorded ICD-10 diagnosis within the F chapter (Mental and behavioral disorders) the month prior to suicide

^c^ At least one recorded ICD-10 diagnosis of ‘R10’, ‘G43’, ‘G44’, ‘R51’, ‘M54’, ‘M25.5’, ‘M79.1’, ‘M79.6’ ‘R52- ‘, ‘R52.9’, ‘M79.7’, ‘R52.1’, ‘R52.2’ or ‘F45.4’ including all subcategories the month prior to suicide

### Differences in proportions consulting health care between suicide cases and the general population of Skåne

**Fig. 1:**
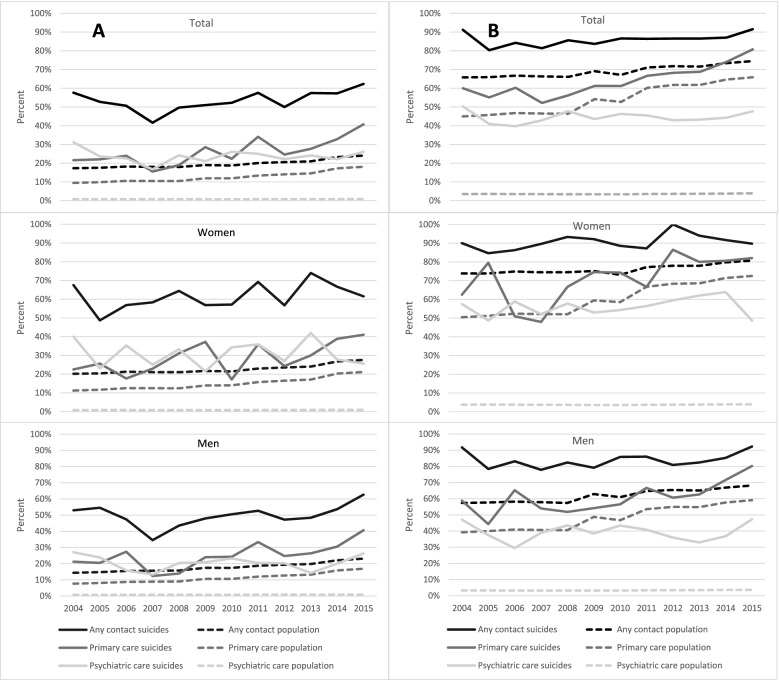
A. Proportion of individuals consulting different types of health care one month prior to suicide and during one month that same year in the general population, total and stratified by women and men, 2004–2015. B. Proportion of individuals consulting different types of health care one year prior to suicide and during that same year in the general population, total and stratified by women and men, 2004–2015

In total, 53% of the suicide victims had at least one health care consultation the month prior to suicide whereas in the general population of Skåne between the years 2004 to 2015 a mean proportion of 20% consulted health care during a one-month period, *p* < 0.0001 (Fig. 1A). For psychiatric care consultations specifically, 24% of the suicide victims as compared to 1% in the general population of Skåne had a consultation, *p* < 0.0001 while 26% of suicide victims and 13% in the general population of Skåne had a health care consultation within primary care, *p* < 0.0001. This pattern was seen among both women and men, although the level of consultations differed between men and women (Fig. 1A).

Looking at the proportion of individuals that had at least one health care contact during the year prior to suicide, the proportion of suicide victims was 86% as compared to 69% in the general population of Skåne *p* < 0.0001 (Fig. 1B). The most noticeable difference was found within psychiatric care where 45% of suicide victims had a health care consultation compared to 4% in the general population of Skåne during a year, *p* < 0.0001. As for primary care consultations, 63% of suicide victims and 54% in the general population of Skåne had at least one health care consultation, *p* < 0.0001. As for the one-month period, the same pattern was seen among both women and men, although also for the one-year period the level of consultations differed between men and women (Fig. 1B).

**Fig. 2:**
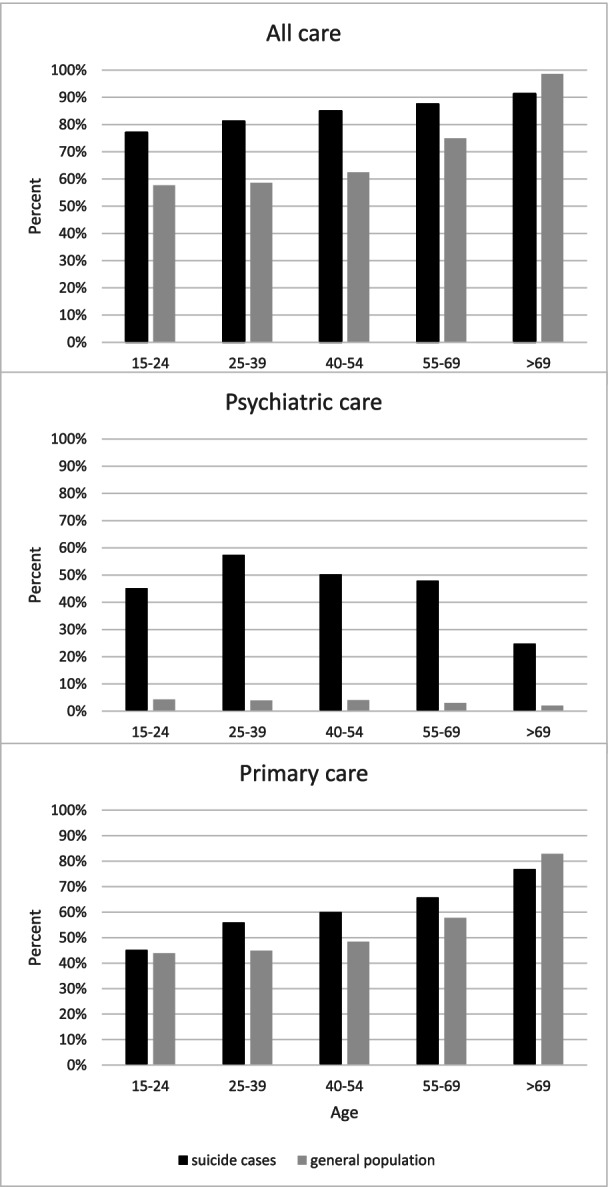
Proportion of individuals consulting different types of health care one year prior to suicide and during that same year in the general population, stratified by age 2004–2015

There was a difference in proportion consulting health care between suicide cases and the general population of Skåne in all age groups, but the pattern varied between types of health care. The largest difference was seen for psychiatric care, Fig. 2.

### Patterns of health care consultations the month preceding suicide

Among those suicide victims that consulted any health care the month prior to the suicide older adults (> 69 years old) were most likely to have any consultation (*p* < 0.001) and a primary care consultation (*p* < 0.001) (Table 2). The ages 25–39 years old were the group most likely to have a psychiatric care consultation (*p* < 0.001). Females were more likely than men to have a consultation in all health care (62% vs. 49%, *p* < 0.001) and in psychiatric care (31% vs. 20%, *p* < 0.001). There was no statistically significant difference regarding consultations in primary care between men and women.

**Table 2:** Proportion consulting health care the one-month preceding suicide, % (n)

	Any health care			Psychiatric care			Primary care		
	Yes	No	*p*-value ^c^	Yes	No	*p*-value	Yes	No	*p*-value
Total	53 (877)	47 (776)		24 (390)	76.4 (1266)		25.8 (427)	74.2 (1226)	
Age			< 0.001			< 0.001			< 0.001
15–24	38 (54)	63 (90)		23 (33)	77 (111)		10 (14)	90 (130)	
25–39	51 (142)	49 (135)		34 (95)	66 (182)		17 (47)	83 (230)	
40–54	48 (200)	52 (220)		26 (107)	75 (313)		21 (87)	79 (333)	
55–69	55 (238)	45 (194)		24 (105)	76 (327)		28 (119)	72 (313)	
≤ 70	64 (243)	36 (137)		13 (50)	87 (330)		42 (160)	58 (220)	
Gender			< 0.001			< 0.001			0.083
Male	49 (563)	51 (580)		20 (232)	80 (911)		25 (281)	75 (862)	
Female	62 (314)	38 (196)		31 (158)	69 (352)		29 (146)	71 (364)	
Psychiatric diagnosis ^a^						< 0.001			< 0.001
Yes	N/A	N/A		77 (358)	23 (105)		40 (187)	60 (276)	
No	N/A	N/A		3 (32)	97 (1158)		20 (240)	80 (950)	
Pain diagnosis ^b^						0.8616			< 0.001
Yes	N/A	N/A		24 (20)	76 (62)		73 (60)	27 (22)	
No	N/A	N/A		24 (370)	76 (1201)		23 (367)	77 (1204)	

^a^ At least one recorded ICD-10 diagnosis within the F chapter (Mental and behavioral disorders) the month prior to suicide

^b^ At least one recorded ICD-10 diagnosis of ‘R10’, ‘G43’, ‘G44’, ‘R51’, ‘M54’, ‘M25.5’, ‘M79.1’, ‘M79.6’ ‘R52- ‘, ‘R52.9’, ‘M79.7’, ‘R52.1’, ‘R52.2’ or ‘F45.4’ including all subcategories the month prior to suicide

^c^ Chi-square test

In total, 463 individuals (28%) had a psychiatric diagnosis registered the month prior to suicide while 82 individuals (3%) had a pain diagnosis during the same period. Individuals with a documented psychiatric diagnosis were more likely than those without to have a consultation in primary care (40% vs. 20%, *p* < 0.001). Individuals with a registered pain diagnosis were more likely than those without to have a consultation in primary care (73% vs. 23%, *p* < 0.001) (Table 2). Notably, almost a quarter (26%) of all suicide victims also had a health care consultation in the week prior to death, 10% in primary care and 9% in psychiatric care (data not shown).

### Patterns of health care consultations the year preceding suicide

Among the suicide victims, consultation rates for any type of health care and for psychiatric care the year before suicide were high and stable throughout the period 2004 to 2015, with no significant time trends in rates of contacts (*p* = 0.053 and *p* = 0.400 respectively).

However, the proportion of individuals having a registered primary care consultation has increased over the time span (*p* < 0.001) (Fig. 1B).

The proportion that consulted any type of health care and primary care increased with age (Table 3). While for psychiatric care, 25–39 years old were the group where the largest proportion that consulted (57%, *p* < 0.001). Females were more likely than men to have both any consultation (91% vs. 84%, *p* < 0.001), psychiatric care consultations (56% vs. 39%, *p* < 0.001) and primary care consultations (70% vs. 60%, *p* < 0.001).

**Table 3:** Proportion consulting health care the one-year preceding suicide, % (n)

	All health care			Psychiatric care			Primary care		
	Yes	No	*p*-value ^c^	Yes	No	*p*-value	Yes	No	*p*-value
Total	86 (1418)	14 (235)		45 (736)	56 (917)		63 (1049)	37 (607)	
Age			< 0.001			< 0.001			< 0.001
15–24	77 (111)	23 (33)		45 (65)	55 (79)		45 (65)	55 (79)	
25–39	81 (225)	19 (52)		57 (159)	43 (118)		56 (155)	44 (122)	
40–54	85 (357)	15 (63)		50 (211)	50 (209)		60 (252)	40 (168)	
55–69	88 (378)	13 (54)		48 (207)	52 (225)		66 (284)	34 (148)	
≤ 70	91 (347)	9 (33)		25 (94)	75 (286)		77 (292)	23 (88)	
Gender			< 0.001			< 0.001			< 0.001
Male	84 (956)	16 (187)		39 (450)	61 (693)		60 (690)	40 (453)	
Female	91 (462)	9 (48)		56 (286)	44 (224)		70 (358)	30 (152)	
Psychiatric diagnosis ^a^						< 0.001			< 0.001
Yes	N/A	N/A		80 (718)	20 (184)		76 (677)	25 (225)	
No	N/A	N/A		2 (18)	98 (733)		49 (371)	51 (380)	
Pain diagnosis ^b^						0.0006			< 0.001
Yes	N/A	N/A		53 (184)	47 (166)		92 (322)	8 (28)	
No	N/A	N/A		42 (552)	58 (751)		56 (726)	44 (577)	

^a^ At least one recorded ICD-10 diagnosis within the F chapter (Mental and behavioral disorders) the year prior to suicide

^b^ At least one recorded ICD-10 diagnosis of ‘R10’, ‘G43’, ‘G44’, ‘R51’, ‘M54’, ‘M25.5’, ‘M79.1’, ‘M79.6’ ‘R52- ‘, ‘R52.9’, ‘M79.7’, ‘R52.1’, ‘R52.2’ or ‘F45.4’ including all subcategories the year prior to suicide

^c^ Chi-square test

In total, 902 individuals (55%) had a psychiatric diagnosis registered the year prior to suicide while 350 (21%) had a registered pain diagnosis. Individuals with a psychiatric diagnosis were more likely than those without to have consultations in primary care (76% vs. 49%, *p* < 0.001). Similarly, individuals with a pain diagnosis were more likely than those without to have a consultation in psychiatric care (53% vs. 42%, *p* = 0.0006) and primary care (92% vs. 56%, *p* < 0.001) (Table 3).

The most frequent mental illness diagnosis registered the year prior to suicide was depression, 23% and anxiety disorder, 12% but also alcohol- and drug use disorders were common, 12% and 7% respectively.

## Discussion

This register-based study that was undertaken in a geographically well-defined region of Sweden, Skåne, shows that health care consultations are common in the near time leading up to suicide. About 53% of the suicide victims had any type of health care consultation one month prior to suicide as compared to the general population of Skåne where 20% had a health care contact during a one-month period. Our findings thus revealed considerable differences in rates of health care use between suicide victims and the general population of Skåne and the patterns varied between women and men and different age groups.

For individuals who died by suicide similar figures are reported from both a Scottish [34] and a recent French study [6] although these studies lack a comparison with the general population. An earlier Swedish study, that did not include primary care data, consequently reported lower overall proportions [3] whereas a study from Wales [25] report higher proportions of health care contacts for both suicide victims and the control population. A Norwegian study reported that 82% consulted their general practitioner within a year of the suicide [22]. This is a substantial difference compared to the proportion that consulted primary care in our study (63%). One reason for the differences could maybe be explained in an actual difference in health care seeking patterns i.e., where you consult, between different countries depending on various factors e.g. organization of and access to health care.

During the one-year period prior to suicide, most individuals in our study had any health care consultation. This is similar to previous studies in various health care settings, including different control samples of non-suicides [4, 8, 22, 25, 35].

Our study confirmed the finding from previous studies that past history of mental illness is an important risk factor for suicide [1, 3, 5] where the proportion of individuals with a previous consultation in psychiatric care was low (4%) in the general population whereas suicide victims had a consultation rate in psychiatric care of 45% the year prior to suicide.

Also, in accordance with previous studies on health care consultation patterns, men were less likely than women to have a consultation prior to suicide [6, 22, 23, 25, 35, 36] while being more likely to actually die by suicide. One possible explanation could be the stigma associated with mental illness and suicide thoughts which is generally higher among men [37, 38]. In line with this, studies have shown that men and women consult health care differently overall and hence in the case of mental illness, such as depression leading to males being under-diagnosed and subsequently under-treated for depression [39–41].

Our study revealed considerable variation in rates of contact by age, both within the group that died from suicide but also as compared to the general population of Skåne. The largest amount of suicides occurred in the ages 40–69 while older adults were most likely to have any type of consultation. This is to be expected due to a higher overall morbidity within the group, but we found that older adults were less likely to have a consultation within psychiatric care. A recent publication from the Swedish Board of health and welfare reports that mental illness is underdiagnosed in the older age group [42], which is also supported by our study. Identifying individuals at risk for suicide is essential for effective suicide prevention. Primary care physicians and other personnel are important in the identification process. Especially so for older adults, who often consult primary care for somatic diseases, but not psychiatric illnesses. Common health problems among the elderly, such as sleep disturbances and loss of appetite, may be misinterpreted as somatic diseases when they could in fact be a sign of mental health issues, for instance depression [43]. Therefore, awareness among primary care physicians is vital as referral to mental health care could reduce suicide rates [44]. A recent Swedish study reported health care deficiencies, which were considered contributing to suicide, in 55% of suicide cases that were reported to the supervisory authority [45]. The most frequent deficiency regarded treatment and suicide risk assessment, further emphasizing the need of a sufficient competence level of mental health issues in health care staff.

Younger individuals had the overall lowest rates of any consultation. Alternative preventive methods should be used to reach younger individuals, especially younger males [22, 46].

We found that more than half of the suicide victims, 55%, had a psychiatric diagnosis and 21% had a pain diagnosis the year leading up to suicide. This was a bit unexpected as other studies have shown that comorbidity between pain and mental illness is common and hence a higher prevalence of pain was expected [16, 47]. However, we only looked at diagnoses the year prior to suicide and nearly four out of ten cases had neither a psychiatric nor a pain diagnosis meaning it is also common for those who die by suicide to seek health care for other conditions i.e. not just psychiatric diseases, which have also been shown in other studies [6]. For those individuals that did have a registered mental illness diagnosis the year prior to the suicide, we found that depression and anxiety were the most prevalent. This is in line with both national [48] and international [5] studies.

A strength of our study is that it uses the total population over several years which allows for a detailed investigation of health care consultation patterns across both sex and age. As earlier mentioned, the two registers included in this study are both considered good, having high quality data and high coverage [29, 30]. Albeit this, some deaths could potentially be misclassified, i.e. registered as accidental deaths but truly being a suicide. A study validating reported cause of deaths in Scandinavia concluded that only few accidents and natural deaths were reclassified as certain suicides, although some degree of under-reporting could not be excluded [49]. Based on these studies therefore, it is reasonable to think that this potential misclassification is so small that it does not change the main findings of our results. Another potential limitation in this study relates to the fact that we only had access to aggregated data for the general population which limited our options for analyses.

On the other hand, we were able to compare suicide victims with the general population using aggregated routinely collected administrative data covering a whole population over twelve years and did so for all types of health care consultations. Our study includes primary care, outpatient care and inpatient care, both for the suicide victims and the general population, and this is not that common among previous studies.

## Conclusions

In conclusion, we show that a majority of people who die by suicide have a previous and close-in-time consultation with health care, hence possible to identify and thus to treat in order to reduce the overall suicide rates. Our findings stress the need of better identification also outside of psychiatric care and especially targeting older people.

## Data Availability

The data included in this study are stored within the Skåne county council. To access similar data, The Swedish National Board of Health and welfare and The Skåne county council can provide information about how to apply for access.

## Abbreviations

ICD: International Classification of Diseases and Related Health Problems
SHR: Skåne healthcare register
